# Using Acidosis as a Surrogate for or Supplement to the Bedside Index of Severity in Acute Pancreatitis Scoring Prediction System Has a Nonsignificant Effect

**DOI:** 10.7759/cureus.63826

**Published:** 2024-07-04

**Authors:** Thomas R Checketts, Suhail Sidhu, Will S Reiche, Ryan W Walters, Haitam Buaisha

**Affiliations:** 1 Internal Medicine, Creighton University School of Medicine, Omaha, USA; 2 Medicine, Creighton University School of Medicine, Omaha, USA; 3 Gastroenterology, Creighton University School of Medicine, Omaha, USA; 4 Clinical Research and Public Health, Creighton University School of Medicine, Omaha, USA

**Keywords:** morbidity in pancreatitis, mortality in pancreatitis, metabolic acidemia, albumin-corrected anion gap, non-anion gap metabolic acidosis, acute pancreatitis, severe pancreatitis

## Abstract

Currently, risk stratification calculators for acute pancreatitis (AP) can at best predict acute pancreatitis mortality at 12 hours from the presentation. Given the severe morbidity associated with AP, the identification of additional prognostic indicators, which may afford earlier prediction in length of stay (LOS) and mortality, is desired. Metabolic acidosis can be a prognostic marker for the severity of AP, and venous bicarbonate can reliably and accurately be substituted for arterial base deficit to detect metabolic acidosis. Since serum bicarbonate, anion gap (AG), and corrected AG (CAG) are routinely obtained upon presentation to the emergency department and often daily in the hospital, we conducted a retrospective analysis of 443 patients, evaluating if venous bicarbonate could predict the severity of pancreatitis as well as mortality, admission to the ICU, ICU LOS, and hospital LOS. The inclusion of venous bicarbonate, AG, and CAG in the first 12 hours only slightly improved the predictive capabilities of the Bedside Index for Severity in Acute Pancreatitis (BISAP) score for these secondary outcomes. None of our incorporations of acidemia improved severity predictions more than the BISAP alone. Adding CAG to BISAP scoring had the largest effect on predicting ICU admission and hospital LOS (area under the curve (AUC): 1.12 (confidence interval (CI) 95%: 1.06-1.19), p <.001 and AUC 1.02 (CI 95% 1.01-1.04), p <.001; respectively). ICU LOS was not impacted by the addition of AG, CAG, or venous bicarbonate. In-hospital death (n=12) was too small to be determined.

## Introduction

Acute pancreatitis (AP) is an inflammatory process that can progress to severe disease with multiorgan failure and death. Rates of AP are increasing throughout the United States [1]. Most patients will develop transient organ disturbances [2]; however, 10-20% of patients will experience severe disease [3-5]. Early recognition and appropriate treatment are essential in the management of this disease. Further studies are needed to predict at an early stage who will develop the severe form of the disease.

Predictive models have incorporated patient demographics, neurologic status, vital signs, lab values, and imaging to determine who will develop severe disease. The Bedside Index of Severity in Acute Pancreatitis (BISAP) is the only scoring system that predicts severity at 12 hours into presentation [6-8], making it superior to Ranson’s score [9], Accuracy of Acute Physiology and Chronic Health Evaluation II (APACHE II) [10], and the Modified Computed Tomography Severity Index (modified CTSI) [11], which use data at the 48-hour mark to prognosticate. Additionally, Ranson’s and APACHE II require arterial blood gas draws, which are not routinely obtained in the work-up of abdominal pain. A prior study of patients with severe AP (SAP) found organ dysfunction may occur earlier than 48 hours in 60% of patients [12]. The BISAP has been examined by several studies showing it to be an accurate predictive scoring mechanism for disease severity [8] and for the risk of mortality in the first 24 hours [7,8,13,14]. Papachristou et al. found the sensitivity of the BISAP score to be 37.5% and the specificity 92.4% for predicting persistent organ dysfunction at 24 hours, with a positive predictive value of 57.7% and a negative predictive value of 84.3% [15]. One meta-analysis found the area under the curve (AUC) for predicting SAP with the BISAP to be 0.77 (95% CI: 0.73-0.80) [14].

Adjusting the BISAP model to allow for even earlier prediction may allow physicians to be proactive in the management of AP and potentially improve outcomes. Various studies in animal and in vitro actually induced acidosis and elevated anion gaps. These studies showed that the presence of acidosis made complications of AP more severe [16-19], may predict acute kidney injury [15], and were a strong independent predictor of severity and mortality. Other animal studies have shown that serum bicarbonate levels may be substituted to detect metabolic acidosis [13,18,20-24]. Serum bicarbonate and anion gap are two ideal objective parameters that are commonly obtained in patients with abdominal pain at the time of initial presentation to the emergency department. The use of values identifying metabolic acidosis may have strong efficacy in these predictions. Additionally, not all patients with AP will receive imaging of the thorax to confirm the presence of pleural effusion. Therefore, we sought to improve upon the predictive capabilities of the BISAP and promote ease by not adding unnecessary tests or imaging to the work-up.

We added initial serum bicarbonate and anion gap values to the BISAP scoring to determine if these scores could improve the predictive capacity of the BISAP for the determination of mild AP, moderate AP, or SAP, the need for ICU admission, ICU LOS, and hospital LOS. Understanding disease severity can guide treatment [25,26]. Other metrics are being studied with more obscure labs (interleukin-6 [27] and microRNA (miR)-155 [28]), which may have a stronger predictive ability than we had. We also tested our models with and without the presence of pleural effusions (BISAP and BISAP without pleural effusion, respectively).

## Materials and methods

Protocol

The study was approved as exempt research by the Institutional Review Board at Creighton University (InfoEd record number: 1433425).

We conducted a retrospective chart review of patients with a primary diagnosis of AP at Catholic Health Initiatives (CHI)-Bergan Mercy Medical Center, a 400-bed academic hospital in Omaha, Nebraska, and its affiliate CHI-Immanuel Medical Center, a 356-bed hospital in Omaha, Nebraska, between June 2017 to June 2019. Patients were included if they were at least 19 years of age (the age of adulthood in Nebraska), were diagnosed with AP within 72 hours of symptom onset, and were managed by CHI’s academic gastroenterology group. AP hospitalizations were identified with the help of an informatician. Once identified, charts were reviewed to assess for complications resulting in organ failures, whether permanent or transient. Some complications included hemoperitoneum, portal vein thrombosis, ileus, complications following ST elevation and non-ST elevation myocardial infarction (NSTEMI) (within a 28-day period). We also evaluated for dependence on renal dialysis, dependence on respirator (ventilator) status, and pancreatic necrosis with or without infection. Patients were excluded if they presented > 72 hours after symptom onset, were treated in another hospital prior to transfer, or had (1) cardiac disease (congestive heart failure (CHF) or symptomatic coronary artery disease (CAD)), (2) malignancy, (3) severe chronic obstructive pulmonary disease with hypercapnia, (4) chronic pancreatitis, (5) diabetic ketoacidosis, or (6) chronic kidney disease (CKD). Patients were also excluded if they were pregnant or managed by a non-academic gastroenterology group. A cutoff criterion of triglycerides > 1000 mg/dL was used for hypertriglyceridemia-induced AP.

For each hospitalization meeting inclusion criteria, we extracted outcomes that included AP severity as per the Revised Atlanta Classification (mild, moderate, or severe) and secondary outcomes that included ICU admission, ICU LOS, and hospital LOS. We extracted patient characteristics, which included age, sex, weight, BMI, days of symptoms prior to presentation, and the presence of comorbidities including diabetes mellitus (DM), chronic kidney disease (CKD), chronic respiratory failure (need for home oxygen), symptomatic coronary artery disease, and chronic pancreatitis. Objective data including patients' systemic inflammatory response syndrome (SIRS) criteria and presence of altered mental status were collected in addition to blood urea nitrogen (BUN), anion gap, serum bicarbonate, and presence of pleural effusion. All patients were given standard medical care throughout the study. BISAP scores were calculated using the first labs obtained in the emergency department. Contrast-enhanced computerized tomography (CECT) was only conducted when indicated. Patients were followed throughout their hospital stay only. The only piece of missing lab data that we encountered was the absence of thoracic imaging to confirm the presence or absence of pleural effusion. When no imaging was available, we assumed that there was no pleural effusion present.

Descriptive statistics for baseline demographic and clinical characteristics were stratified by AP severity. Continuous variables are presented as mean and standard deviation or median and interquartile range, with between-group comparisons evaluated using either one-way analysis of variance (ANOVA) or the Kruskal-Wallis test. Categorical variables are presented as count and percent, with comparisons evaluated using the chi-square test or Fisher’s exact test. The discriminant abilities of BISAP and BISAP without pleural effusion scores, with or without bicarbonate or anion gap (original or corrected), to differentiate between mild AP, moderate AP, and SAP were evaluated using the area under the curve (AUC) estimated by a cumulative logit regression model. Higher AUC values indicate better discriminant ability. The 95% confidence interval for each AUC was calculated based on 1,000 bootstrapped replications; statistical comparisons of AUC were based on the mean and standard deviation across the bootstrapped replications.

To avoid the assumption of equal weighting or contribution of individual components of the BISAP or BISAP without pleural effusion score, AUCs were based on models that included each component separately. When including bicarbonate or anion gap, we used restricted cubic splines to evaluate whether the association with AP severity was linear with pre-specified knot points at the fifth, 35th, 65th, and 95th percentiles. The incremental benefit of serum bicarbonate, anion gap, and corrected anion gap for ICU admission, ICU LOS, and hospital LOS were assessed using sequentially estimated models with between-model comparisons conducted using the likelihood ratio test. Logistic regression models were estimated for ICU admission, whereas negative binomial regression models were estimated for ICU LOS (for patients in the ICU) and hospital LOS. All models controlled for biological sex, race, BMI, and the severity of AP. All analyses were conducted using SAS version 9.4 (SAS Institute, Cary, North Carolina, United States), with two-tailed p < .05 used to indicate statistical significance.

## Results

A total of 443 patients were included in the analysis with a mean age of 48.4 years (range: 19-91), 50.8% female, and 74.8% White. Of these patients, 316 (71.3%) were classified with mild AP, 93 (21.0%) were classified with moderate AP, and 34 (7.7%) were classified with SAP (Table 1). The majority of AP was due to gallstone or idiopathic pancreatitis. Patients with SAP were older and had higher baseline creatinine, albumin, or hemoglobin.

**Table 1:** Demographic, clinical, and laboratory characteristics stratified by acute pancreatitis severity Data presented as mean ± standard deviation, median (interquartile range), or count (percent) LOS: length of stay

Characteristic	Mild (n = 316)	Moderate (n = 94)	Severe (n = 34)	p-value
Age	48.4 ± 17.1	52.4 ± 17.6	57.4 ± 14.5	0.004
Race				
Hispanic, African American, and Asian American	79 (27.3)	20 (23.0)	4 (12.5)	0.161
White	210 (72.7)	67 (77.0)	28 (87.5)	
Biological sex				
Female	170 (53.8)	37 (39.8)	11 (32.4)	0.007
Male	146 (46.2)	56 (60.2)	23 (67.7)	
BMI	28.8 ± 8.6	28.7 ± 8.2	30.0 ± 7.5	0.727
Etiology				
Alcohol	109 (34.5)	37 (39.8)	14 (41.2)	
Gallstone	96 (30.4)	26 (28.0)	11 (32.4)	0.548
Hypertriglyceridemia	7 (2.2)	5 (5.4)	1 (2.9)	
Idiopathic	104 (32.9)	25 (26.9)	8 (23.5)	
Laboratory				
Albumin	3.6 (3.3-3.9)	3.4 (3.0-3.9)	3.3 (2.9-3.9)	< .001>
Chloride	105 (101-107)	102 (98-106)	104 (98-108)	0.105
Creatinine	0.9 (0.7-1.1)	0.9 (0.8-1.1)	1.2 (1.0-1.4)	< .001>
Hemoglobin	14.0 (12.4-15.2)	14.1 (12.6-16.0)	14.6 (13.2-16.4)	0.049
Lipase (per 1000)	4.2 (2.1-11.0)	4.2 (2.1-9.6)	7.0 (4.2-21.2)	0.068
Sodium	139 (136-140)	138 (133-140)	139 (135-141)	0.115
Total LOS	3 (2-5)	5 (3-8)	7 (6-14)	< .001>
ICU admittance	22 (7.0)	17 (18.3)	18 (52.9)	< .001>
ICU LOS	4 (3-7)	5 (3-7)	8 (5-15)	0.014
In-hospital death	1 (0.3)	2 (2.2)	9 (26.5)	< .001>

**Figure 1:**
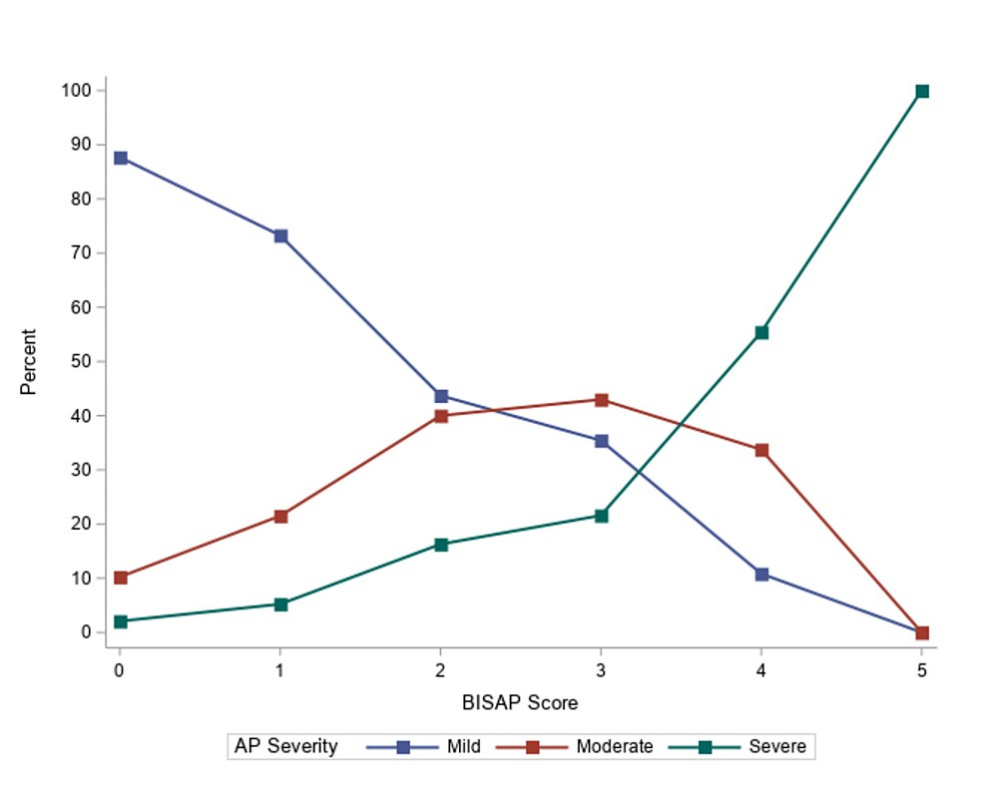
Bedside Index of Severity in Acute Pancreatitis (BISAP) score and severity AP: acute pancreatitis

BISAP data are presented in Table 2. As expected, higher BISAP scores were associated with greater AP severity (Figure 1). Serum bicarbonate was statistically lower in patients with greater AP severity. The discriminant ability of BISAP and BISAP without pleural effusion scores, with or without bicarbonate and anion gap, are presented in Table 3. Both BISAP and BISAP without pleural effusion had an acceptable discriminant ability for AP severity, although, there were benefits including pleural effusions. The highest AUCs were associated with BISAP scores that included anion gap, whether original (AUC: 0.77, 95% CI: 0.72 to 0.82) or corrected (AUC: 0.78, 95% CI: 0.73 to 0.82). No AUC was statistically different from BISAP.

**Table 2:** Bedside Index of Severity in Acute Pancreatitis (BISAP) characteristics Data presented as mean ± standard deviation, median (interquartile range), or count (percent) BUN: blood urea nitrogen; SIRS: systemic inflammatory response syndrome

Characteristic	Mild (n = 316)	Moderate (n = 94)	Severe (n = 34)	p-value
BISAP	1 (0-1)	1 (1-2)	2 (2-3)	< .001>
0	154 (48.7)	21 (22.6)	1 (2.9)	< .001>
1	117 (37.0)	39 (41.9)	5 (14.7)	
2	32 (10.1)	26 (28.0)	13 (38.2)	
3	12 (3.8)	5 (5.4)	9 (26.5)	
4	1 (0.3)	2 (2.2)	4 (11.8)	
5	0 (0.0)	0 (0.0)	2 (5.9)	
BISAP components				
BUN > 25 mg/dL	20 (6.3)	11 (11.8)	15 (44.1)	< .001>
Impaired mental status	7 (2.2)	6 (6.5)	11 (32.4)	< .001>
≥2 SIRS criteria	117 (37.0)	55 (59.1)	28 (82.4)	< .001>
Age > 60 years	77 (24.4)	26 (28.0)	17 (50.0)	0.006
Pleural effusion	9 (2.9)	20 (21.5)	14 (41.2)	< .001>
Laboratory				
Bicarbonate	24.4 ± 4.0	23.7 ± 4.7	22.4 ± 6.3	0.032
Anion gap				
Original	9 (7-11)	10 (8-12)	11 (9-14)	0.009
Corrected	10 (8-12)	11 (10-14)	14 (12-16)	< .001>

**Table 3:** Area under the curve (AUC) for the revised Atlanta classification of acute pancreatitis All analyses included individual components of the Bedside Index of Severity in Acute Pancreatitis (BISAP) with and without pleural effusion. For the AUC, higher values indicate better discrimination. All p-values are relative to the BISAP score.

Characteristic	AUC (95% CI)	p-value
BISAP	0.76 (0.71-0.80)	-
BISAP + bicarbonate	0.77 (0.72-0.81)	0.847
BISAP + anion gap		
Original	0.77 (0.72-0.82)	0.658
Corrected	0.78 (0.73-0.82)	0.587
BISAP without pleural effusion	0.71 (0.66-0.76)	0.149
BISAP without pleural effusion + bicarbonate	0.72 (0.66-0.77)	0.244
BISAP without pleural effusion + anion gap		
Original	0.73 (0.68-0.78)	0.385
Corrected	0.74 (0.69-0.78)	0.503

Greater odds of admission to the ICU were associated with SAP and the presence of at least two SIRS criteria (Table 4). When considering the addition of serum bicarbonate and anion gap, the best model fit was indicated for corrected anion gap; although, all three measures were statistically associated with admission to the ICU. Specifically, a one-unit higher corrected anion gap was associated with a 12% greater adjusted odds of an ICU admission (95% CI: 5% to 19% greater, p < .001; Table 4, Model 5), a one-unit higher anion gap was associated with a 10% greater adjusted odds of an ICU admission (95% CI: 4% to 17% greater, p = .002), and a one-unit higher serum bicarbonate was associated with a 9% lower adjusted odds of an ICU admission (95% CI: 2% to 16% lower, p = .011). Further, for the 57 patients admitted to the ICU, longer ICU LOS was associated with greater AP severity (Table 5); however, serum bicarbonate and anion gap were not associated with ICU LOS (Table 5; Models 3-5).

**Table 4:** Admission to ICU Any adjusted odds ratio (aOR) greater than 1 indicates greater odds of ICU admission. For biological sex, race, and acute pancreatitis severity, the reference group is identified after the “vs.” For example, for Model 1, patients with severe acute pancreatitis had 12.22 greater odds of ICU admission compared to patients with mild acute pancreatitis. Bicarbonate, anion gap, and corrected anion gap are continuous variables, so the aORs represent higher/lower odds of an ICU admission per one-unit increase in bicarbonate, anion gap, or corrected anion gap. For example, for Model 5, every one-unit higher corrected anion gap was associated with a 12% higher odds of ICU admission (i.e., (1.12 – 1)*100). Overall, models with a lower Akaike information criterion (AIC) fit the data better. Here, the model with the corrected anion gap is the best. BISAP: Bedside Index for Severity in Acute Pancreatitis; BUN: blood urea nitrogen; SIRS: systemic inflammatory response syndrome; -2LL: negative 2 log-likelihood

	Model 1		Model 2		Model 3		Model 4		Model 5	
Covariates	aOR (95% CI)	p-value	aOR (95% CI)	p-value	aOR (95% CI)	p-value	aOR (95% CI)	p-value	aOR (95% CI)	p-value
Male vs. female	1.61 (0.81-3.21)	0.067	1.61 (0.81-3.21)	0.178	1.71 (0.84-3.46)	0.138	1.57 (0.77-3.18)	0.215	1.58 (0.78-3.22)	0.207
White vs. Hispanic, African American, and Asian American	0.49 (0.23-1.03)	0.177	0.48 (0.23-1.02)	0.057	0.57 (0.26-1.25)	0.161	0.57 (0.26-1.24)	0.156	0.55 (0.25-1.20)	0.136
BMI	0.97 (0.92-1.01)	0.220	0.97 (0.92-1.01)	0.136	0.97 (0.93-1.02)	0.266	0.99 (0.94-1.04)	0.649	0.99 (0.95-1.04)	0.758
AP severity										
Severe vs. mild	12.22 (3.90-38.31)	< .001	11.06 (3.38-36.21)	< .001	10.76 (3.23-35.88)	< .001	10.24 (3.09-33.89)	< .001	9.13 (2.73-30.52)	< .001
Severe vs. moderate	5.53 (1.74-17.57)	0.004	5.25 (1.65-16.75)	0.005	5.37 (1.65-17.45)	0.005	5.03 (1.57-16.13)	0.007	4.74 (1.46-15.33)	0.010
Moderate vs. mild	2.21 (1.03-4.71)	0.041	2.11 (0.96-4.61)	0.062	2.00 (0.91-4.43)	0.086	2.03 (0.91-4.52)	0.082	1.93 (0.86-4.31)	0.110
BISAP components										
BUN > 25 mg/dL	0.55 (0.16-1.89)	0.344	0.57 (0.17-1.95)	0.370	0.68 (0.20-2.31)	0.541	0.64 (0.19-2.13)	0.466	0.67 (0.20-2.24)	0.515
Impaired mental status	3.72 (1.23-11.30)	0.020	3.61 (1.19-10.99)	0.024	2.34 (0.71-7.75)	0.164	1.89 (0.56-6.33)	0.305	1.73 (0.5-5.82)	0.380
≥2 SIRS criteria	6.18 (2.66-14.39)	< .001	6.08 (2.61-14.15)	< .001	5.67 (2.42-13.31)	< .001	5.80 (2.44-13.78)	< .001	5.81 (2.42-13.94)	< .001
Age > 60 years	0.72 (0.32-1.65)	0.442	0.71 (0.31-1.63)	0.425	0.78 (0.34-1.82)	0.570	0.95 (0.41-2.23)	0.909	0.92 (0.40-2.15)	0.848
Pleural effusion			1.28 (0.50-3.25)	0.606	1.29 (0.50-3.34)	0.602	1.43 (0.55-3.69)	0.466	1.38 (0.53-3.56)	0.508
Serum bicarbonate					0.91 (0.84-0.98)	0.011				
Anion gap							1.10 (1.04-1.17)	0.002		
Anion gap - corrected									1.12 (1.05-1.19)	< .001
Model fit parameters										
-2LL	197.6		195.9		182.5		179.2		176.8	
AIC	217.6		217.9		206.5		203.2		200.8	
p-value (to Model 2)	-		-		< .001		< .001		< .001	

**Table 5:** ICU length of stay Any adjusted rate ratio (aRR) greater than 1 indicates a longer ICU length of stay. For biological sex, race, and acute pancreatitis severity, the reference group is identified after the “vs.” For example, for Model 1, patients with severe acute pancreatitis had a 76% longer ICU length of stay compared to patients with mild acute pancreatitis (i.e., (1.76 – 1)*100). Bicarbonate, anion gap, and corrected anion gap are continuous variables, so the aRRs represent higher/lower ICU length of stay per one-unit increase in bicarbonate, anion gap, or corrected anion gap. For example, for Model 5, every one-unit higher corrected anion gap was associated with a 2% longer ICU length of stay (i.e., (1.02 – 1)*100). Overall, models with a lower Akaike information criterion (AIC) fit the data better. Here, the model with the corrected anion gap is best, but not by much. BISAP: Bedside Index for Severity in Acute Pancreatitis; BUN: blood urea nitrogen; SIRS: systemic inflammatory response syndrome; -2LL: negative 2 log-likelihood

	Model 1		Model 2		Model 3		Model 4		Model 5	
Covariates	aRR (95% CI)	p-value	aRR (95% CI)	p-value	aRR (95% CI)	p-value	aRR (95% CI)	p-value	aRR (95% CI)	p-value
Male vs. female	0.81 (0.61-1.08)	0.144	0.79 (0.58-1.06)	0.114	0.82 (0.60-1.12)	0.200	0.81 (0.60-1.10)	0.173	0.81 (0.60-1.09)	0.162
White vs. Hispanic, African American, and Asian American	1.28 (0.91-1.80)	0.158	1.31 (0.92-1.86)	0.129	1.33 (0.93-1.88)	0.110	1.32 (0.93-1.87)	0.119	1.33 (0.94-1.88)	0.107
BMI	0.98 (0.96-1.01)	1.131	0.98 (0.96-1.00)	0.107	0.98 (0.96-1.01)	0.151	0.99 (0.96-1.01)	0.229	0.99 (0.97-1.01)	0.287
AP severity										
Severe vs. mild	1.76 (1.20-2.59)	0.005	1.84 (1.23-2.74)	0.004	1.81 (1.22-2.68)	0.004	1.80 (1.21-2.69)	0.005	1.76 (1.18-2.63)	0.007
Severe vs. moderate	1.61 (1.11-2.33)	0.013	1.62 (1.12-2.34)	0.012	1.55 (1.07-2.26)	0.022	1.55 (1.07-2.26)	0.023	1.52 (1.04-2.23)	0.032
Moderate vs. mild	1.10 (0.76-1.57)	0.611	1.13 (0.78-1.65)	0.502	1.16 (0.80-1.69)	0.415	1.16 (0.80-1.69)	0.421	1.16 (0.80-1.68)	0.428
BISAP components										
BUN > 25 mg/dL	0.67 (0.45-0.99)	0.049	0.64 (0.41-0.98)	0.040	0.70 (0.44-1.12)	0.133	0.68 (0.43-1.08)	0.099	0.69 (0.44-1.09)	0.110
Impaired mental status	1.15 (0.84-1.59)	0.375	1.17 (0.85-1.62)	0.327	1.10 (0.78-1.55)	0.584	1.06 (0.73-1.55)	0.742	1.04 (0.71-1.52)	0.822
≥2 SIRS criteria	1.05 (0.69-1.62)	0.808	1.09 (0.70-1.69)	0.697	1.11 (0.71-1.72)	0.648	1.15 (0.73-1.82)	0.529	1.16 (0.74-1.83)	0.512
Age > 60 years	0.99-0.71-1.37)	0.936	1.02 (0.72-1.45)	0.888	1.10 (0.76-1.58)	0.612	1.11 (0.76-1.61)	0.590	1.12 (0.77-1.63)	0.543
Pleural effusion			0.89 (0.60-1.32)	0.545	0.88 (0.59-1.30)	0.508	0.90 (0.61-1.34)	0.604	0.90 (0.61-1.34)	0.605
Serum bicarbonate					0.99 (0.96-1.01)	0.276				
Anion gap							1.01 (0.99-1.04)	0.324		
Anion gap - corrected									1.02 (0.99-1.05)	0.253
Model fit parameters										
-2LL	230.8		230.4		229.2		229.4		229.1	
AIC	252.8		254.4		255.2		255.4		255.1	
p-value (to Model 2)	-		-		0.273		0.317		0.254	

Finally, longer hospital LOS was associated with greater AP severity, BUN greater than 25 mg/dL, having at least two SIRS criteria, and the presence of pleural effusion (Table 6). The addition of corrected anion gap or serum bicarbonate improved model fit (Table 6; Models 3 and 5) with a one-unit higher corrected anion gap associated with a 2% longer hospital LOS (95% CI: 1% to 3% longer, p = .032) and a one-unit higher bicarbonate associated with a 2% shorter hospital LOS (95% CI: 1% to 4% shorter, p = .021).

**Table 6:** Hospital length of stay Any adjusted rate ratio greater than 1 indicates a longer hospital length of stay. For biological sex, race, and acute pancreatitis severity, the reference group is identified after the “vs.” For example, for Model 1, patients with severe acute pancreatitis had 2.6 times longer hospital length of stay compared to patients with mild acute pancreatitis. Bicarbonate, anion gap, and correct anion gap are continuous variables, so the aRRs represent higher/lower hospital length of stay per one-unit increase in bicarbonate, anion gap, or corrected anion gap. For example, for Model 5, every one-unit higher corrected anion gap was associated with a 2% longer hospital length of stay (i.e., (1.02 – 1)*100). Overall, models with a lower Akaike information criterion fit the data better. Here, the model with the corrected anion gap is the best. BISAP: Bedside Index for Severity in Acute Pancreatitis; BUN: blood urea nitrogen; SIRS: systemic inflammatory response syndrome; -2LL: negative 2 log-likelihood

	Model 1		Model 2		Model 3		Model 4		Model 5	
Covariates	aRR (95% CI)	p-value	aRR (95% CI)	p-value	aRR (95% CI)	p-value	aRR (95% CI)	p-value	aRR (95% CI)	p-value
Male vs. female	0.86 (0.74-0.98)	0.026	0.86 (0.75-0.98)	0.028	0.87 (0.76-1.00)	0.054	0.86 (0.75-0.98)	0.027	0.86 (0.75-0.99)	0.031
White vs. Hispanic, African American, and Asian American	0.98 (0.84-1.15)	0.823	0.97 (0.82-1.13)	0.672	1.00 (0.85-1.18)	0.980	1.00 (0.85-1.17)	0.963	1.00 (0.85-1.17)	0.955
BMI	0.99 (0.98-0.99)	0.010	0.99 (0.98-0.99)	0.022	0.99 (0.98-1.00)	0.071	0.99 (0.98-1.00)	0.089	0.99 (0.98-1.00)	0.111
AP severity										
Severe vs. mild	2.62 (2.03-3.38)	< .001	2.28 (1.75-2.96)	< .001	2.25 (1.73-2.92)	< .001	2.25 (1.73-2.93)	< .001	2.21 (1.70-2.88)	< .001
Severe vs. moderate	1.55 (1.18-2.02)	0.002	1.47 (1.12-1.92)	0.005	1.47 (1.13-1.92)	0.005	1.47 (1.13-1.92)	0.005	1.45 (1.11-1.90)	0.006
Moderate vs. mild	1.69 (1.43-2.00)	< .001	1.55 (1.31-1.84)	< .001	1.53 (1.29-1.81)	< .001	1.53 (1.30-1.82)	< .001	1.52 (1.29-1.80)	< .001
BISAP components										
BUN > 25 mg/dL	1.39 (1.11-1.74)	0.004	1.39 (1.11-1.74)	0.004	1.42 (1.14-1.77)	0.002	1.41 (1.13-1.76)	0.003	1.42 (1.14-1.78)	0.002
Impaired mental status	1.34 (0.99-1.81)	0.059	1.26 (0.93-1.70)	0.131	1.14 (0.84-1.55)	0.410	1.16 (0.85-1.58)	0.357	1.14 (0.84-1.56)	0.399
≥2 SIRS criteria	1.27 (1.10-1.47)	0.001	1.25 (1.08-1.44)	0.002	1.22 (1.06-1.41)	0.005	1.23 (1.07-1.42)	0.004	1.23 (1.06-1.41)	0.005
Age > 60 years	1.03 (0.88-1.21)	0.678	1.03 (0.88-1.20)	0.735	1.05 (0.89-1.22)	0.574	1.06 (0.90-1.24)	0.500	1.05 (0.90-1.23)	0.541
Pleural effusion			1.49 (1.20-1.86)	< .001	1.50 (1.21-1.86)	< .001	1.50 (1.21-1.86)	< .001	1.48 (1.19-1.84)	< .001
Serum bicarbonate					0.98 (0.96-0.99)	0.021				
Anion gap							1.01 (0.99-1.03)	0.061		
Anion gap - corrected									1.02 (1.01-1.03)	0.032
Model fit parameters										
-2LL	1955.4		1942.3		1937.0		1938.8		1937.6	
AIC	1977.4		1966.3		1963.0		1964.8		1963.6	
p-value (to Model 2)	-		-		0.021		0.061		0.030	

## Discussion

Our review of 443 patients with AP found serum bicarbonate and anion gap did not increase the predictive value of the BISAP score for differentiating between mild AP, moderate AP, and SAP. Additionally, the prediction of admission to the ICU and hospital LOS were associated with the use of bicarbonate, anion gap, and corrected anion gap. The addition of these values improved the predictive capabilities, but not in a statistically significant manner. ICU LOS could not be determined due to the small sample size.

It is important to diagnose pancreatitis early as hemoconcentration and impaired pancreatic microcirculation have a high mortality rate when patients are under-resuscitated. The early stratification of severity also determines if more intensive monitoring in the ICU is necessary. Most research agrees with our findings that meeting ≥ 2 SIRS criteria is an accurate predictor of the need for ICU admission. It can also help predict the likelihood of intra-abdominal infections, though not directly observed in our data.

One of the key aspects of our study was to explore enhancements and modifications to the BISAP score that can be readily calculated with as routine work-up as possible. We ventured to compare our findings, which replaced thoracic imaging with surrogates, showing that acidemia (anion gap and correct anion gap) would affect the score. Overall, our results were not unlike those of other studies. Our AUC for the BISAP alone was 0.76 (95% CI: 0.71-0.80) and for the BISAP + CAG was 0.78 (95% CI: 0.73-0.82), which was not very different from the population-based study of 17,992 patients by Wu et al., which found the BISAP AUC to be 0.82 (95% CI: 0.79 to 0.84) [28]. Yang and Li did a meta-analysis that included 1,972 patients and had a pooled AUC of 0.77 (95% CI: 0.73-0.80) [14]. These suggest that our population was like those of these reported studies.

The efforts we took to add to the score were in fact comparable to other studies using unmodified scoring including BISAP, Ranson’s, and APACHE II. Harshit and Singh did a comparison study of CTSI, Ranson’s, Apache II, and BISAP. They also evaluated BISAP’s ability to predict SAP with an AUC of 0.684 (95% CI: 0.518-0.849, n = 31) and ICU admission with an AUC of 0.877 (95% CI: 0.739-1.000, n = 14) [11]. BISAP alone had an AUC of 0.76 (95% CI: 0.71-0.80), whereas BISAP + CAG had a slightly higher AUC of 0.78 (95% CI: 0.73-0.82). The difference in AUC was not statistically significant (p = 0.587). Overall, the AUCs found in these studies were like those found with BISAP + corrected anion gap and BISAP without pleural effusion + corrected anion gap, our most indicative results. Papachristou et al. examined the efficacy of BISAP compared to Ranson’s, Apache II, and CTSI in the stratification of severity with 185 patients. They found their BISAP AUC to be 0.81 (95% CI: 0.74-0.87) [15]. The substitutions, exclusions, and inclusions of our study provided only marginal benefit and in a non-statistically significant manner.

Our BISAP manipulations produced AUC results within a range of 0.71 (the BISAP without pleural effusion and BISAP without pleural effusion + bicarbonate) to 0.78 (BISAP + corrected anion gap). However, these ranges were not significantly different from one another. Ours is not the first study to add additional laboratory values to the BISAP score. In addition to the BISAP score, Wu et al. incorporated miR-155 values to show much stronger predictive abilities to calculate SAP than BISAP alone with an AUC of 0.945 (95% CI: 0.931-0.959) [28]. Though these innovative tests are promising, they are not available at most hospitals. Their inclusion broadly would add delays to stratification and extra costs. Testing for miR is increasingly being studied in the context of disease for its role in assessing inflammation. Given the inability to have early and affordable disease stratification, we do not advocate for its use universally. As mentioned earlier, our purpose was not to find the best test for severity stratification but to better utilize readily obtained labs to make triage decisions for patients.

There were limitations to the study. Our sample size of in-hospital mortality (n=12) was too limited to be used for predictive scoring purposes. A low mortality rate may have demonstrated the acuity of patients or reflected the appropriateness of early treatment. Given that our study was a retrospective chart review, patient identification was closely tied to diagnostic coding, which may not accurately capture all diagnostic criteria indicating disease and/or severity. There were missing data that were not obtained from these charts; however, most missing data were tied to missing chest X-rays, which are not as commonly obtained in patients with abdominal pain as compared to a basic metabolic panel (BMP) and a complete blood count (CBC). We accounted for this through chart checking the images obtained in the first 12 hours of admission. When a patient did not have a chest X-ray, we assumed that they did not have a pleural effusion, which represents another limitation. The distinction between types of crystalloid solution was not made; although lactated ringers (LRs) have been found to be superior to normal saline, we were not able to ensure all patients included in the analysis received LRs.

Pancreatitis is a disease with significant morbidity and mortality. Early disease severity stratification treatment is essential to prevent deterioration. Unfortunately, there are few scoring metrics in use that can accurately stratify patients into groups based on future likelihood of being mild, moderate, and/or severe. This study contains important findings on how systemic acidosis has moderate efficacy in predicting disease severity in pancreatitis, but replication is required using data from other health systems to confirm. Given the numerous attempts of previous scores that prognosticate mortality and/or morbidity at 48 hours and beyond, it is important for further research to be done examining at which point acidosis becomes relevant for disease severity. Additional data are required to identify which other routine and readily available labs can be used early in admission or patient encounters to stratify patients into disease severity cohorts.

## Conclusions

Pancreatitis is a disease with significant morbidity and mortality that remains difficult to stratify early and with routine laboratory metrics. Earlier stratification can assist providers in triaging severity and managing resources appropriately for care. This retrospective review of a single center found that adding surrogate markers reflecting acidemia and metabolic acidosis was not statistically different than the use of the BISAP alone. 
